# Effects of FMO3 Polymorphisms on Pharmacokinetics of Sulindac in Chinese Healthy Male Volunteers

**DOI:** 10.1155/2017/4189678

**Published:** 2017-02-26

**Authors:** Yong-Jun Tang, Kai Hu, Wei-Hua Huang, Chong-Zhi Wang, Zhi Liu, Yao Chen, Dong-Sheng Ouyang, Zhi-Rong Tan, Hong-Hao Zhou, Chun-Su Yuan

**Affiliations:** ^1^Department of Clinical Pharmacology, Xiangya Hospital, Central South University, Changsha 410008, China; ^2^Institute of Clinical Pharmacology, Hunan Key Laboratory of Pharmacogenetics, Central South University, Changsha 410078, China; ^3^Department of Pediatrics, Xiangya Hospital, Central South University, Changsha 410008, China; ^4^Department of Neurology, Xiangya Hospital, Central South University, Changsha 410078, China; ^5^Tang Center for Herbal Medicine Research, The Pritzker School of Medicine, University of Chicago, 5841 South Maryland Avenue, MC 4028, Chicago, IL 60637, USA

## Abstract

Sulindac is a nonsteroidal anti-inflammatory drug, which is clinically used for the ailments of various inflammations. This study investigated the allele frequencies of FMO3 E158K and E308G and evaluated the influences of these two genetic polymorphisms on the pharmacokinetics of sulindac and its metabolites in Chinese healthy male volunteers. Eight FMO3 wild-type (FMO3* HHDD*) subjects and seven FMO3 homozygotes E158K and E308G mutant (FMO3* hhdd*) subjects were recruited from 247 healthy male volunteers genotyped by PCR-RFLP method. The plasma concentrations of sulindac, sulindac sulfide, and sulindac sulfone were determined by UPLC, while the pharmacokinetic parameters of the two different FMO3 genotypes were compared with each other. The frequencies of FMO3 E158K and E308G were 20.3% and 20.1%, respectively, which were in line with Hardy-Weinberg equilibrium (*D*′ = 0.977, *r*^2^ = 0.944). The mean values of *C*_max_, AUC_0–24_, and AUC_0–∞_ of sulindac were significantly higher in FMO3* hhdd* group than those of FMO3* HHDD* group (*P* < 0.05), while the pharmacokinetic parameters except *T*_max_ of sulindac sulfide and sulindac sulfone showed no statistical difference between the two groups. The two FMO3 mutants were in close linkage disequilibrium and might play an important role in the pharmacokinetics of sulindac in Chinese healthy male volunteers.

## 1. Introduction

**Figure 1:**
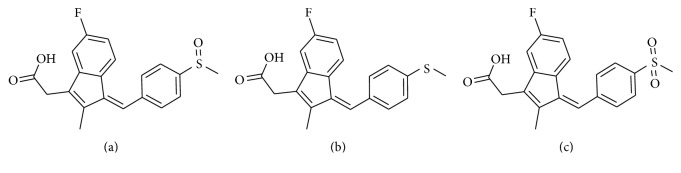
Chemical structure of sulindac (a) and its metabolites and sulindac sulfide (b) and sulindac sulfone (c).

Sulindac, approved by FDA in 1978, is a nonsteroidal anti-inflammatory drug (NSAID), which has clinically been used for the ailments of various inflammations over thirty years [1]. NSAIDs have antipyretic, analgesic, and anti-inflammatory effects by blocking the synthesis of prostaglandins due to the significant inhibition of cyclooxygenase [2]. Sulindac as a prodrug that contains a racemic sulfoxide moiety could be transformed into the active drug, sulindac sulfide, by gut microbiota before absorption, while it could be metabolized by CYP450 in liver after absorption [3]. Subsequently, sulindac sulfide is oxidized to be the inactive metabolite, sulindac sulfone (Figure 1), which is catalyzed by the principal isozyme involved in the main metabolic pathway, flavin-containing monooxygenase subtype 3 (FMO3) [3, 4]. The normal product of FMO3 contains 532 amino acids (Molecular weight,* ca.* 60 kDa) [5, 6], which is responsible for the oxidation of many clinically important drugs such as cimetidine [7], ranitidine [8], benzydamine [9], clozapine [10], and sulindac [11].

To date, approximate forty single nucleotide polymorphisms (SNPs) have been identified in the FMO3 gene, ten of which cause altered enzyme activity and may affect its substrates metabolism [12]. FMO3/Lys^158^ and FMO3/Gly^308^ have been identified as two predominant FMO3 genetic mutants, which could inactivate the enzyme and the polymorphisms of which appeared at approximately 18% of both mutations in Korean population [13]. In vivo assay demonstrated that the FMO3 activity showed no difference between heterozygous and homozygous volunteers carrying either one of the two alleles, while its activity would be significantly diminished in the subjects who were either heterozygous or homozygous with both alleles [13]. Additionally, it was reported that a mild symptom of trimethylaminuria (TMAU) could be triggered by a TMA challenge test in patients who were homozygous with FMO3 Lys^158^/Gly^308^ [14]. Therefore, the activity of FMO3 may be weakened among individuals who are homozygous or heterozygous with both mutations.* S*-oxygenation of sulindac sulfide was more significantly inhibited by methimazole in human liver microsomes genotyped as homozygous FMO3 [Glu158Lys; Glu308Gly] gene compared to that genotyped as wild-type or heterozygote. As well, the tendency was observed in the recombinant including wild-type and FMO3 [Glu158Lys; Glu308Gly] genes [15, 16]. To date, it is unknown whether FMO3 polymorphisms have effects on pharmacokinetics of sulindac in Chinese population.

In this study, we aimed to investigate the allele frequencies of FMO3 E158K and E308G in Chinese healthy population and evaluate the effects of the two FMO3 genetic polymorphisms on pharmacokinetics of sulindac as well as its metabolites in Chinese healthy male volunteers.

## 2. Experimental

### 2.1. Subjects

The subjects were restricted to be between 18 and 23 years old with standard body mass index between 20 and 25 kg/m^2^. Fifteen healthy male subjects (age: 20.5 ± 2.1 years; height: 175.5 ± 8.6 cm; weight: 65.5 ± 5.5 kg.) were recruited in this study according to the clinical protocol. All the healthy subjects were requested to sign the informed consent after the assessments of physical examination, medical history, electrocardiogram, and standard laboratory tests including blood cell, urinalysis, and biochemical profile. All of the volunteers were required to abstain from alcohol and medication and could only take standard diets during the study. The subjects who had taken antibiotics in three weeks before this study could not be recruited. The study protocol was approved by the Ethics Committee of Xiangya School of Medicine, Central South University, Hunan province, China.

### 2.2. Study Design

This project was an open-label, randomized, and single-period study. Human plasma was collected from healthy male subjects who were recruited in this pharmacokinetic study. The test drug named Zulida (sulindac tablets; 100 mg per tablet; batch number 060301) was supplied by Ningbo Team Pharm Co., Ltd. (Hangzhou, China). After fasting overnight, each subject was administered with a single oral dose of sulindac tablets (200 mg) with 100 mL water. Meanwhile, 5 mL of venous blood samples was collected into an EDTA (K2)-containing tubes from a forearm vein immediately before dosing (0 h) and at 10 min, 20 min, 30 min, 45 min, 1 h, 1.5 h, 2 h, 4 h, 6 h, 8 h, 10 h, 12 h, 24 h, and 36 h after drug administration. The blood samples were centrifuged at 3000 RPM for 10 min. The collected plasma samples were stored at −40°C until analysis. The subjects were allowed to have meal in 4 h after oral administration with sulindac tablets.

### 2.3. Genotyping

In order to screen FMO3 E158K and E308G polymorphisms in this study, 247 healthy Chinese male volunteers were tested to find the subjects in the defined genotypes. Consequently, fifteen healthy male Chinese subjects were totally enlisted, including eight homozygous subjects with the wild-type allele (FMO3* HHDD*) and seven homozygous volunteers with the mutant allele (FMO3* hhdd*).

**Figure 2:**
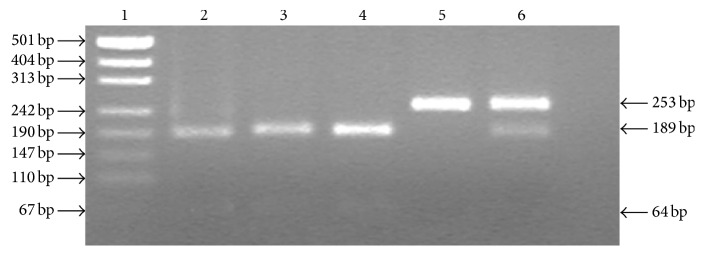
Agarose gel electrophoresis of FMO3 E158K after PCR-RFLP. DNA 500 bp marker ladder (Lane 1); wild homozygous, PCR products digested to 189 bp and 64 bp band (Lanes 2, 3, and 4); mutant homozygote (Lane 5); heterozygote (Lane 6).

**Figure 3:**
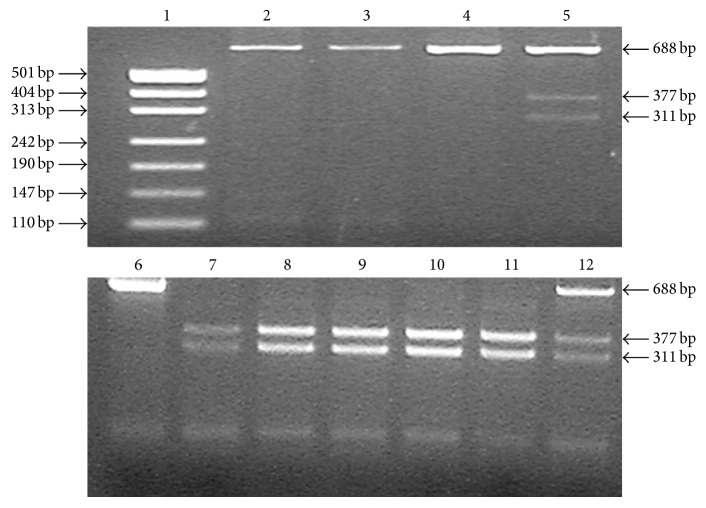
Agarose gel electrophoresis of FMO3 E308G after PCR-RFLP. DNA 500 bp marker ladder (Lane 1); heterozygote (Lanes 2, 3, 4, and 6); mutant homozygote, PCR products digested to 377 bp + 311 bp band (Lanes 7, 8, 9, 10, 11, and 12).

The FMO3/Lys^158^ allele was assigned by the lowercase “h” which was identified by the HinFI restriction enzyme while the FMO3/Glu^158^ was assigned by the corresponding uppercase “H”; the FMO3/Gly^308^ allele was assigned by the lowercase “d” which was identified by DraII, and the FMO3/Glu^308^ was designated by an uppercase “D”; genomic DNA was extracted from 5 mL peripheral blood by standard phenol-chloroform method. The wild-type allele (FMO3* HHDD*) and the homozygous mutant allele (FMO3* hhdd*) were identified according to polymerase chain reaction-restriction fragment length polymorphism (PCR-RFLP) as described previously with slight modification [17, 18]. The PCR-RFLP experimental conditions were provided in Table 1, and the Macrorestriction Maps of FMO3 E157K and E308G were shown in Figures 2 and 3, respectively.

### 2.4. Analytical Assay

Plasma concentrations of sulindac and its metabolites, sulindac sulfide and sulindac sulfone, were quantified by ultraperformance liquid chromatography tandem Photodiode Array (UPLC-PDA) [19]. The analytes were directly extracted by dichloromethane from the collected human plasma using a liquid-liquid extraction method. The chromatographic separation was achieved on Waters Acquity UPLC installed with a Waters Acquity UPLC BEH C18 column (2.1 × 50 mm i.d., 1.7 *μ*m) within 5 minutes. The mobile phase consisting of ammonium formate buffer (20 mM) containing 1% acetic acid and acetonitrile was used for gradient elution. The flow rate was 0.4 mL/min and the monitor wavelength for PDA detection was set at 328 nm.

### 2.5. Statistical Analysis

The areas under the plasma concentration-time curve (AUC) of sulindac, sulindac sulfide, and sulindac sulfone were calculated according to the linear trapezoidal rule. The elimination rate constant (*K*_*e*_) was calculated by linear regression of the terminal points using the semilog plot of plasma concentration versus time. The half-life of elimination (*T*_1/2_) was calculated by using the formula *T*_1/2_ = 0.693/*K*_*e*_. The area under the plasma concentration-time curve (AUC_0–36_) was calculated by using the linear trapezoidal rule. The area under the plasma concentration-time curve to time infinity (AUC_0–*∞*_) was yielded from the calculations: AUC_0–*∞*_ = AUC_0–36_ + *C*_36_/*K*_*e*_, in which *C*_36_ was the pose-dose plasma concentration of the drug at 36 h.

The pharmacokinetic parameters were calculated by Drug and Statistics for Windows (DAS ver1.0) software. Data were presented as mean ± SE or SD of at least three independent experiments performed for each sample analysis. The pharmacokinetic parameters between the two genotyped groups were evaluated by Student's *t*-test. Data analysis was performed by the SPSS 19.0 software for windows (Chicago, IL, USA). Values of *P* < 0.05 were considered to be statistically significant.

## 3. Results

Before the pharmacokinetics study, previous data showed that the frequency of the FMO3* hhdd* genotype was 0.046 in Korean populations, much higher than that in White populations [20]. In this study, we found that FMO3 E158K and E308G were in close linkage disequilibrium in Chinese and the LD between these two SNPs were as strong as that in Koreans. Meanwhile, it was reported that FMO3* hhdd* genotype has a frequency of 0.047 in Chinese populations similar to those previously calculated in Korean populations [21]. Under the lifestyle education, our pilot observations on the subjects with the two different genotypes [21] showed that the effective rate was approximately 80%. Thus, we need to enroll 212 subjects to ensure an 80% power to detect that the drug group has significant effects compared to that of wild-genotype volunteers. Considering factors such as dropouts, 247 subjects were recruited for the screening, while eight homozygous subjects with the wild-type allele (FMO3* HHDD*) and seven homozygous volunteers with the mutant allele (FMO3* hhdd*) were subjected in this study.

Our previous report on the determination of sulindac and its metabolites, sulindac sulfide and sulindac sulfone, in human plasma had demonstrated that the developed method was precise, accurate, and stable for quantification of these analytes [19]. The extraction efficiencies of sulindac, sulindac sulfide, and sulindac sulfone were all >75%. The upper limits of quantification were 104.1 *μ*g/mL for sulindac, 117.6 *μ*g/mL for sulindac sulfide, and 104.4 *μ*g/mL for sulindac sulfone in this assay. The lower limits of quantification were 813.4 ng/mL, 918.8 ng/mL, and 815.6 ng/mL for sulindac, sulindac sulfide, and sulindac sulfone, respectively. The analytical recoveries for all the three analytes were in the range of 85%–110%, while the interday and intraday precision for the three analytes were all less than 15%.

Significant linkage disequilibrium (LD) was apparent between the two alleles in Chinese population (*D*′ = 0.9774, *r*^2^ = 0.9442, Table 2). Eight subjects with FMO3* HHDD* genotype and seven subjects with FMO3* hhdd* genotype were randomly chosen and enrolled in the pharmacokinetic study of sulindac. With a single 200 mg oral dose of sulindac tablets, the pharmacokinetic parameters of sulindac were significantly different between FMO3* HHDD* homozygote and FMO3* hhdd* homozygote. AUC_0–*∞*_ value of sulindac in the FMO3* hhdd* group was much higher than that in the FMO3* HHDD* group (41.88 ± 17.37 versus 27.93 ± 8.85 *μ*g·h/mL, *P* < 0.05). *C*_max_ value of sulindac in FMO3* hhdd* group was higher than that in FMO3* HHDD* group (11.87 ± 4.80 versus 6.95 ± 3.28 *μ*g/mL, *P* < 0.05). *T*_1/2_ value of sulindac FMO3* hhdd* group was higher than that in FMO3* HHDD* group (4.46 ± 1.30 versus 4.01 ± 1.24 h, *P* < 0.05). Meanwhile, *C*_max_, *T*_1/2_, AUC_0–24 h_, and AUC_0–*∞*_ of sulindac sulfide and sulindac sulfone in FMO3* hhdd* group were not significantly lower than those in the FMO3* HHDD* group. The main pharmacokinetic parameters of sulindac, sulindac sulfide, and sulindac sulfone after oral administration of 200 mg sulindac tablets were also calculated, while the main pharmacokinetic parameters of sulindac, sulindac sulfide, and sulindac sulfone in different genotypes were provided in Table 3.

**Figure 4:**
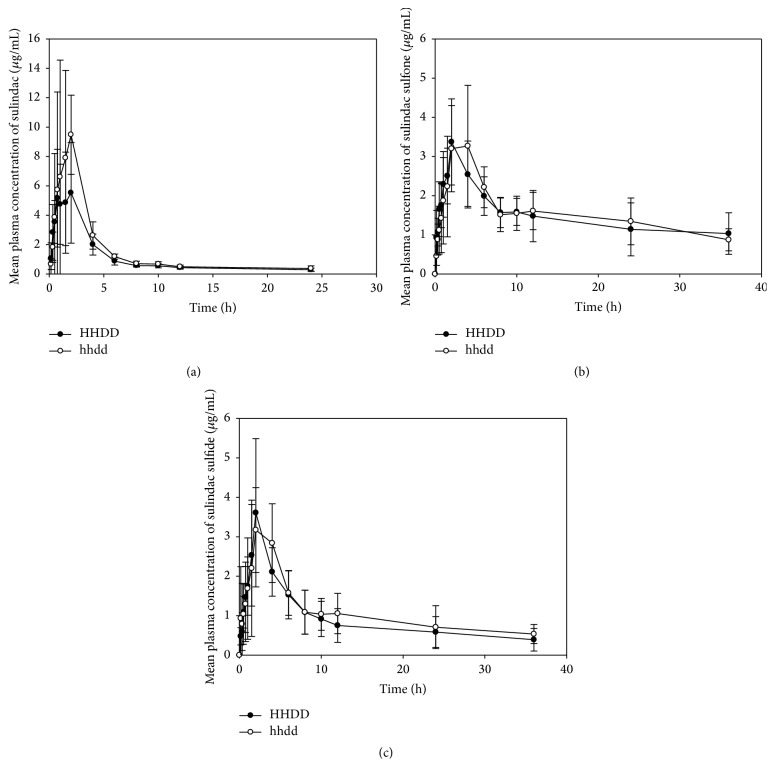
The mean plasma concentration-time curves of sulindac (a), sulindac sulfide (b), and sulindac sulfone (c) in FMO3* HHDD* homozygotes (*n* = 8) and FMO3* hhdd* homozygotes (*n* = 7) after a single oral dose of 200 mg sulindac tablets.

The mean plasma concentration-time curves of sulindac, sulindac sulfide, and sulindac sulfone were plotted. The mean plasma concentration-time curves for sulindac, sulindac sulfide, and sulindac sulfone in FMO3* HHDD* group and FMO3* hhdd* group were described in Figure 4.

## 4. Discussion

In this study, 247 Chinese healthy volunteers were recruited for FMO3* hhdd* genotype analysis. Previous data showed that the frequency of the FMO3* hhdd* genotype was 0.046 in Korean populations, which was much higher than that in White populations [20]. In Mongolian race, it was the first time to discover that LD between FMO3 E158K and E308G in Chinese was as strong as Koreans. Fortunately, we identified thirteen subjects with the FMO3* hhdd* genotype with a frequency of 0.047 that was similar to those previously reported in Korean populations [20].

The effects of genetic polymorphisms of FOM3 on substrates metabolism had already been reported such as the Michaelis constant (*K*_*m*_) for trimethylamine, which was cautiously increased in the homozygous FMO3 Lys^158^ mutants compared with that in the wild-type carriers [22]. Additionally, individuals with heterozygous or homozygous Lys^158^/Gly^308^ alleles had significantly decreased the plasma concentration of trimethylamine* N*-oxygenation after oral administration of trimethylamine [14]. Moreover, both mutant alleles had been found in patients with trimethylaminuria, who had difficulties in metabolizing trimethylamine [23]. Hereby, it was reasonably concluded that the genetic polymorphisms of FMO3 would have effects on the metabolizing of clinical drugs as FMO3's substrates.

NSAIDs including sulindac are effectively chemopreventive agents in some autosomal dominant genetic diseases [24], such as familial adenomatous polyposis (FAP) with an approximate incidence rate of 1/10,000, in which a great number of adenomatous polyps are generated mainly in the epithelium of the large intestine [25]. Sulindac is a prodrug that is converted by gut microbacteria or CYP450 into its active metabolite, sulindac sulfide, which could be catalyzed into its inactive product by FMO3 [11, 26]. To date, no study pertinent to the pharmacokinetics of sulindac, sulindac sulfide, and sulindac sulfone associated with FMO3 genotype in healthy subjects has been reported so that FMO3 should be considered to be the critical factor for the clinical effect of sulindac [27, 28]. Importantly, the FMO3 SNPs of E158K and E308G mutations were able to significantly increase the efficacy of sulindac.

This study evidenced that FMO3 polymorphism might have a considerable impact on the pharmacokinetics of sulindac, rather than that of sulindac sulfide and sulindac sulfone. FMO3* HHDD* and* hhdd* groups exhibited significantly different pharmacokinetic parameters of sulindac. *C*_max_, *T*_max_, AUC_0–24_, and AUC_0–*∞*_ of sulindac were much higher in the seven homozygous FMO3* hhdd* subjects than those in the eight homozygous FMO3* HHDD* subjects (*P* < 0.05), while differences regarding *C*_max_, *T*_1/2_, AUC_0–24_, and AUC_0–*∞*_ of sulindac sulfone and sulindac sulfide were not significant in these two groups. *T*_max_ of sulindac sulfide was lower in FMO3* hhdd* subjects than in FMO3* HHDD* subjects (*P* < 0.05). No significant difference was observed in the relative *T*_max_ values of sulindac sulfone between the two genotyped groups, because sulindac sulfone was absorbed from gastrointestinal tract to keep away from the influence of FMO3. The exposure of sulindac was definitely higher in FMO3* hhdd *homozygous subjects than in FMO3* HHDD* homozygous subjects, which was clearly discriminated by the remarkable discrepancy that AUC_0–*∞*_ value in FMO3* hhdd* carriers was much higher. The results indicated that mutants with FMO3* hhdd *genotype could decrease FMO3 enzyme activity, which could change the in vivo biotransformation of sulindac. Though the mutants showed the tendency to reduce the metabolism of sulindac sulfide and sulindac sulfone as manifested by the data of AUC and *C*_max_, the differences were not statistically significant. The actual effects of different FMO3 genotypes on sulindac sulfide and sulindac sulfone need further study, because the secondary metabolism and transportation on the two metabolites were unknown and the sample size was limited in this study.

The FMO3 homozygous Lys^158^/Gly^308^ carriers had diminished the enzyme activity in catabolizing sulindac. Similar changes were also observed in the pharmacokinetics of benzydamine and ranitidine [20]. It was concluded that FMO3 mutant allele coding proteins with impaired activity ultimately resulted in changes of the clinical consequence for FMO3 substrates. Therefore, the higher plasma concentration of sulindac was possible to increase the toxicity and side effects in FMO3* hhdd* subjects. The drug-drug interactions with sulindac should be cautiously considered, when sulindac is coadministered with other FMO3 substrates in patients carrying the FMO3* hhdd* mutant.

## 5. Conclusion

FMO3 E158K and E308G polymorphisms played an important role in in vivo metabolism of sulindac. The plasma concentration of sulindac was remarkably higher in the individuals carrying FMO3* hhdd* allele than that of the individuals with FMO3* HHDD* (wild-type) allele. More attention should be notably paid when sulindac is coadministered with other FMO3 substrates in patients carrying FMO3* hhdd* mutant.

## Figures and Tables

**Table 1:** PCR-RFLP experiment design of E158K and E308G.

Genetic variance	Primers	Restriction endonuclease	Alleles
15167G>A (E158K)	5′-CTGTCTTTGATGCTGTAATGG-3′	HinfI	H, h
	5′-CAGAAGCGACTGTGAATAG-3′		
21443A>G (E308G)	5′-AACAGGGAACTGGGCATAAG-3′	DraII (EcoO109I)	D, d
	5′-ATTGTCACTGGCATTCATCTTC-3′		

**Table 2:** Analysis of linkage disequilibrium of E158K and E308G.

Genotype	Number	(%)
HHDD	174	(63.5)
HHDd	2	(0.7)
HHdd	0	(0)
HhDD	3	(1.7)
HhDd	82	(29.9)
Hhdd	0	(0)
hhDD	0	(0)
hhDd	0	(0)
hhdd	13	(4.7)
Total	247	(100.0)
*D*′	0.977	
*r* ^2^	0.944	

*D*′ > 0.5 and *r*^2^ > 1/3, which showed linkage disequilibrium. (http://analysis.bio-x.cn/myAnalysis.php).

**Table 3:** Mean pharmacokinetic parameters of sulindac, sulindac sulfide, and sulindac sulfone for FMO3 *HHDD* (*n* = 8) and FMO3 *hhdd* (*n* = 7).

	*FMO3 HHDD*	*FMO3 hhdd*
Sulindac		
AUC_0–*∞*_ (ng·h·mL^−1^)	27.93 ± 8.85	41.88 ± 17.37^*∗*^
AUC_0–24 h_ (ng·h·mL^−1^)	22.82 ± 7.87	35.16 ± 9.60^*∗*^
*C*_max_ (ng·mL^−1^)	6.95 ± 3.28	11.87 ± 4.80^*∗*^
*T*_max_ (h)	1.82 ± 1.07	1.64 ± 0.48
*T*_1/2_ (h)	4.01 ± 1.24	4.46 ± 1.30^*∗*^
Sulindac sulfide		
AUC_0–*∞*_ (ng·h·mL^−1^)	43.69 ± 15.23	49.67 ± 25.92
AUC_0–24 h_ (ng·h·mL^−1^)	32.93 ± 15.59	32.22 ± 13.56
*C*_max_ (ng·mL^−1^)	32.93 ± 15.59	32.22 ± 13.56
*T*_max_ (h)	2.43 ± 1.59	3.07 ± 1.17^*∗*^
*T*_1/2_ (h)	10.18 ± 3.28	9.77 ± 3.97
Sulindac sulfone		
AUC_0–*∞*_ (ng·h·mL^−1^)	65.64 ± 16.98	65.69 ± 24.34
AUC_0–24 h_ (ng·h·mL^−1^)	48.18 ± 0.31	42.76 ± 14.67
*C*_max_ (ng·mL^−1^)	3.43 ± 1.19	3.69 ± 1.61
*T*_max_ (h)	2.57 ± 0.98	2.86 ± 1.07
*T*_1/2_ (h)	12.19 ± 2.44	10.62 ± 2.31

*C*_max_, maximum plasma concentration; AUC, area under plasma concentration-time curve; *T*_max_, time of maximum plasma concentration; *T*_1/2_, half-life of elimination. ^*∗*^*P* < 0.05.
